# microRNA-630 Regulates Underglycosylated IgA1 Production in the Tonsils by Targeting TLR4 in IgA Nephropathy

**DOI:** 10.3389/fimmu.2020.563699

**Published:** 2020-11-26

**Authors:** Chan Liu, Mu-Yao Ye, Wen-Zhe Yan, Xiao-Fei Peng, Li-Yu He, You-Ming Peng

**Affiliations:** ^1^International Medical Department, The Second Xiangya Hospital, Central South University, Changsha, China; ^2^Department of Nephrology, The Second Xiangya Hospital, Central South University, Changsha, China; ^3^Department of Hematology, The Second Xiangya Hospital, Central South University, Changsha, China; ^4^Department of Rheumatology and Immunology, The Second Xiangya Hospital, Central South University, Changsha, China

**Keywords:** IgA nephropathy, palatal tonsils, microRNA-630, underglycosylated IgA1, TLR4

## Abstract

IgA nephropathy (IgAN) is the most common primary glomerular disease. The characteristic pathology involves immune complexes formed by the deposition of IgA1 and underglycosylated IgA1 aggregates in the mesangial area, which may be accompanied by the deposition of IgG and/or IgM and complement components. However, the molecular mechanisms of IgAN remain unclear. In the present study, microarray analysis showed that the expression of microRNA-630 (miR-630) was significantly reduced in palatal tonsils from IgAN patients compared with chronic tonsillitis. Additionally, bioinformatic analysis showed that Toll-like receptor 4 (TLR4) was the predicted target gene of miR-630 and was regulated by miR-630. When miR-630 was overexpressed in palatal tonsil mononuclear cells from IgAN patients, the expression of TLR4 was reduced and the content of IgA1 in the cell culture supernatant was decreased, and the level of galactosylation in the IgA1 hinge region was increased. Moreover, immunohistochemical analysis showed that the expression of TLR4 in IgAN patients was significantly increased. After knocking down the expression of TLR4, both the concentration of IgA1 and the binding force of IgA1 with broad bean lectin were significantly reduced in IgAN. Furthermore, the mechanism study demonstrated that TLR4 might regulate the expression of IL-1β and IL-8 through NF-κB signaling pathway to modulate the concentration of IgA1 and the glycosylation level of IgA1. This interesting finding may offer new insight into the molecular mechanism of IgAN.

## Introduction

IgA nephropathy (IgAN) is considered the most common form of primary glomerulonephritis throughout the world (1), and more than 40% patients with IgAN develop into end-stage renal disease (ESRD) within 20–25 years (2). The main clinical manifestations of IgAN are hematuria, proteinuria, and progressive renal dysfunction (3). The main pathological characteristic is immune complexes formed by the deposition of IgA1 and underglycosylated IgA1 aggregates in the mesangial area (4, 5). However, the molecular mechanism of IgA is still poorly understood.

Recently, studies have confirmed that IgAN is closely related to tonsil mucous membrane immune dysfunction. IgA deposition in the glomerular mesangial region of IgAN patients mainly involves J-chain positive underglycosylated IgA1, which was accordance with the production of underglycosylated IgA1 by palatal tonsil mononuclear cells in IgAN patients (6–9). In addition, our previous study also demonstrated that serum IgA levels were significantly lower in tonsillectomized cases compared to non-tonsillectomized patients; hematuria as well as proteinuria were also decreased greatly (10, 11). These data suggested that the deposition of underglycosylated IgA1 in the mesangial region of IgAN was partially produced by the tonsil. Furthermore, the percentage of memory B cells in tonsils could predict the progression of IgAN (12, 13). Therefore, focusing on the study of palatal tonsil immune response disorders in IgAN may be helpful to elucidate the mechanism of the production of underglycosylated IgA1 in IgAN patients.

MicroRNAs (miRNAs) are small non-coding RNAs with a length of 19~23bp that negatively regulate gene expression post-transcriptionally by targeting the 3’-untranslated regions (3’-UTR) of protein-coding messenger RNA (mRNA) transcripts; they play important roles in different physiological and pathological processes (14, 15). Recently, studies have shown that miRNAs also participate in regulating IgAN (14, 16, 17). For example, Serino *et al*. found that miR-148 could promote the production of underglycosylated IgA1 by down-regulating the expression of C1GALT1 (16). Another study showed that miR-100-3p and miR-877-3p regulated the overproduction of IL-8 and IL-1β in mesangial cells activated by secretory IgA from IgAN patients (17). Nevertheless, the role of miRNAs expressed in palatal tonsils in IgAN pathogenesis is poorly investigated.

In the present study, we evaluated the miRNA expression profiles of tonsils from IgAN and chronic tonsillitis patients and found that the expression of miR-630 was significantly reduced in tonsils from IgAN patients (IgAN group) compared with those from chronic tonsillitis patients (CT group). Furthermore, the mechanistic study demonstrated that Toll-like receptor 4 (TLR4) was the target gene of miR-630 and it was involved in modulating the content of underglycosylated IgA1 by regulating the expression of IL-1b and IL-8 through NF-kB signaling pathway in cell culture supernatants. Therefore, miR-630 may be a non-invasive biomarker used to diagnose and predict the prognosis of IgAN and can be regarded as a potential pharmacologic target for the treatment of IgAN.

## Material and Methods

### Patients and Tonsil Tissue Samples

In the present study, 27 tonsil tissues from IgAN patients (IgAN group) and 20 tonsil tissues from patients with chronic tonsillitis (CT group), who all underwent palatal tonsillectomy under general anesthesia, were collected from the Department of Otorhinolaryngology of the Second Xiangya Hospital, Central South University. All of the IgAN cases were diagnosed by symptoms, clinical examination, and renal biopsy. The patients were not treated with angiotensin-converting enzyme inhibitors (ACEI), angiotensin receptor blocker (ARB), glucocorticoids, or immunosuppressants. Microcirculation drugs were stopped 3 days before tonsillectomy. For the CT group, the renal function of the patients was normal without hematuria or albuminuria. Patients with secondary nephropathy were excluded, including those with systemic lupus erythematosus, Henoch-Schonlein purpura, hepatic disease, diabetic nephropathy, and so on. The clinical data of the participants are shown in **Supplemental Table 1**. The study was conducted in accordance with the Code of Ethics of the World Medical Association (Declaration of Helsinki) and approved by the Ethics Committee of Second Xiangya Hospital, Central South University. All participants gave their written informed consent.

### MicroRNA Microarray Analysis

The miRNA microarray analysis of tonsil tissues was done by Shanghai Bohao Biotechnology Co., Ltd. Briefly, total RNA was extracted with the mirVana™ miRNA Isolation Kit (AM1560, Ambion, Austin, TX) and was fluorescently labeled with the Agilent miRNA chip kit (Agilent Technologies, Santa Clara, CA). Then, RNA was subjected to rolling hybridization at 20 rpm for 20 h at 55°C. After the hybridization was completed, the tablets were washed. The results were scanned with an Agilent microarray scanner and read with Feature Extraction software 10.7. The data were standardized and subjected to cluster analysis to obtain a heat map of the differential genes. The IgAN group/CT group >2 was selected as up-regulated genes, and the IgAN group/CT group <0.5 was regarded as down-regulated genes.

### Cell Culture and Transfection

Tonsil tissues from the IgAN group and CT group were soaked in PBS for 15 min, fully ground, and filtered through a 200 mesh sieve, then the filtrate was collected. Slowly, an equal volume of lymphocyte separation solution was added (Solarbio Technology Co. Beijing, China) and centrifuged at 1,500 rpm for 20 min to retain the middle mononuclear cell layer, and then inactivated 2% fetal bovine serum (FBS, Gibco, Invitrogen) was added. After washing with PBS three times, the cells were resuspended and centrifuged at 1,500 rpm for 5 min. Finally, the tonsil mononuclear cells (TMCs) were cultured in RPMI-1640 medium with 10% FBS, L-glutamine (2 mmol/L), penicillin (100 μg/ml), streptomycin (100 μg/ml) at 37°C in a humidified atmosphere with 5% CO_2_. For miR-630 mimic transfection, TransIT-TKO transfection reagent was used and transfected according to the manufacturer’s instructions. Briefly, 25 nM of miR-630 mimic with FAM-labeled (GenePharma, Shanghai, China), 4 µl of TransIT-TKO transfection reagent and 50 µl of Opti-MEM Reduced Medium (Invitrogen, USA) was incubated for 20 min and then mixed with the TMCs derived from one IgAN patient per well. A miRNA negative control was also transfected at the same time. The TMCs were incubated for 48–72 h at 37°C in 5% CO_2_ and used for total RNA and protein extraction.

### Quantitative Reverse Transcription-Polymerase Chain Reaction (qRT-PCR)

Total RNA was extracted from cultured TMCs by using TRIzol Reagent (Invitrogen, 15596-026), and the All-in-One™ miRNA qRT-PCR Detection Kit (AOMD-Q060, GeneCopoeia, German) and All-in-One™ qPCR Mix Kit (QP002, GeneCopoeia, German) were used to detect the expression of miRNAs and genes [C1GALT1, Cosmc, B cell activating factor (BAFF), B cell activating factor receptor (BAFF-R), IL-1β, IL-8]. First, the first-strand complementary DNA (cDNA) was reverse transcribed at 37°C for 60 min followed by 85°C for 5 min. The cDNA was diluted five-fold before use in the PCR reaction, and then quantitative PCR was performed on an ABI Prism 7300 Sequence Detection System (Applied Biosystems, Foster City). The reaction conditions were 95°C for 10 min followed by 40 cycles at 95°C for 10 s, 57°C for 20 s, and 72°C for 15 s. Relative quantification of miRNA and genes levels was performed with the CT value, which was normalized to the expression of the human U6 gene or GAPDH in each sample using the standard curve method (2^−△△CT^). Each sample was measured in triplicate. The primer sequence of these genes (C1GALT1, Cosmc, BAFF, BAFF-R, IL-1β, IL-8, GAPDH) were shown in the **Supplemental Table 2**.

### Different Subtypes of Mononuclear Cells Isolation and Total RNA Extraction

Different subtypes of mononuclear cells in the tonsil of each participant were separated by gradient centrifugation at 400 × g for 30 min at 4°C and enriched using magnetic beads. In brief, the tonsil tissues were cut into small pieces and ground gently with the plunger of a 5 ml syringe until single-cell suspensions were obtained. Samples were filtered through nylon mesh to remove debris, then the tonsil cell suspensions were centrifuged at 300 × g for 10 min at 4°C. The erythrocytes in the cell suspensions were eliminated using a lysis solution (Solarbio, Beijing, China). The CD4^+^ T cells, CD8^+^ T cells, B cells and monocytes from the tonsil single-cell suspensions were isolated by using the MojoSort™ Human CD4 T cell, CD8 T cell, pan B cell, and Pan Monocyte Cell Isolation Kit (Cat No. 480009, 480011, 480081, 480059, BioLegend Inc. San Diego, CA, USA), respectively, according to the manufacturer’s instructions.

### Luciferase Reporter Assay

Vectors bearing the wild type TLR4 3’ UTR (psiCHECK2-TLR4) or the 3’ UTR with mutations at the putative binding site of miR-630 (psiCHECK2-TLR4-mutmiR-630) were constructed. TMCs were seeded in 24-well plates and, 24 h later, 500 ng of reporter plasmid was cotransfected with 100 pmol of miR-630 mimic or negative control (GenePharma) using Lipofectamine 3000. Cells were collected 48 h after transfection and luciferase activity was detected *via* a luciferase reporter assay system (Promega) according to the manufacturer’s protocols.

### Western Blotting

Total proteins were extracted by radio immunoprecipitation assay (RIPA), EDTA-free protease inhibitor and phosphatase inhibitor PhosSTOP (RIPA: protease inhibitor: phosphatase inhibitor=98:1:1) (Beyotime Biotechnology, Shanghai, China). The protein was quantified using the BCA Protein Kit (Beyotime Biotechnology, Shanghai, China). Forty micrograms of protein samples were submitted to sodium dodecyl sulfate polyacrylamide *gel* electrophoresis (SDS-PAGE) electrophoresis and transferred to a PVDF membrane (Millipore, Billerica, MA, USA) with a transfer membrane apparatus (Bio-Rad, USA). The membrane was then blocked with 5% BSA (Sigma) for 1 h and incubated with primary antibodies at 4°C overnight. The antibodies included TLR4 (sc-293072, 1:500, Santa Cruz Biotechnology), C1GALT1 (ab237734, 1:1,000, Abcam), Cosmc (ab229831, 1:1,000, Abcam), BAFF (ab224710, 1:1,000, Abcam), B cell activating factor receptor BAFF-R (ab233775, 1:1,000, Abcam), NF-κB p65 (ab16502, 1:1,000, Abcam), and β-actin (66009-1-Ig, 1:5,000, Proteintech). The next day, the membrane was washed with PBS-T three times for 15 min, followed by incubation with HRP-labeled goat anti-mouse (sc-2005, 1:4,000, Santa Cruz Biotechnology) or HRP-labeled goat anti-rabbit (074-1506, 1:3,000, KPL) secondary antibody at 37°C for 1 h. Luminata™ Crescendo Western HRP Substrate (200~300 µl) was added and then the images were developed. After obtaining the results, the images were processed using Kodak MI software, and the gray values of the bands were evaluated and analyzed using Image J software. The relative expression level of the target protein was normalized to the intensity of the β-actin band.

### Immunohistochemistry

Tonsil tissue samples were fixed and processed by the paraffin-embedded method. Tonsil tissue sections were deparaffinized in xylene and rehydrated in a graded ethanol series. The sections were incubated with 3% hydrogen peroxide in order to clear endogenous peroxidase and followed by antigen retrieval with trypsin. After incubation with 5% BSA (100 µl per well) at 37°C for 30 min, the sections were incubated with TLR4 primary antibody (1:100, Santa Cruz) at 4°C overnight. The next day, sections were washed with PBS three times and incubated with HRP-labeled secondary antibody (1:2,000, Beyotime Institute of Biotechnology) at 37°C for 1 h. Positive staining was revealed using glucose oxidase diaminobenzidine (DAB). The images were acquired from a fluorescence microscope (Nikon) and analyzed with Image-Pro Plus Version 6.0. All immunohistochemical experiments were repeated at least three times and representative images are presented.

### ELISA

The supernatant of the TMCs was collected and 96-well immunoplates (Costar, Cambridge, MA, USA) were coated with mouse anti-human IgA1 antibody (1:400, Santa Cruz Biotechnology) at 4°C overnight. Plates were washed five times with PBS containing 0.05% Tween-20, then blocked with PBS containing 1% BSA at 37°C for 2 h. Next, supernatant samples or standard human IgA1 (Calbiochem, La Jolla, CA., USA) were added to each well (100 µl) and incubated at 37°C for 2 h. The plates were washed five times, then biotin-labeled mouse anti-human IgA1 (1:2,000, Southern Biotechnology) was added to each well (100 µl) and incubated at 37°C for 1 h. The plates were washed five times, and HRP-labeled streptavidin (1:4,000, Beyotime Institute of Biotechnology) was added to each well (100 µl) and incubated at 37°C for 30 min. Finally, 0.1 mg/ml of tetramethylbenzidine (TMB) was added at room temperature for 5 min. The optical density (OD) was measured at 450 nm. Each sample was assayed in duplicate and repeated more than three times.

### *Vicia villosa* Lectin Binding Assay

The *Vicia villosa* lectin (VVL) binding assay was used to measure the binding of the O-glycan–specific lectin from VV to antigen-immobilized IgA1 using our previously mentioned ELISA method. VVL recognizes terminal O-linked GalNAc, and IgA1 samples with lower terminal galactosylation show a higher lectin binding force. The supernatant of TMCs was collected and 96-well immunoplates (Costar, Cambridge, MA, USA) were coated with mouse anti-human IgA1 antibody (1:400, Santa Cruz Biotechnology) at 4°C overnight. Plates were washed five times with PBS containing 0.05% Tween-20, then blocked with PBS containing 1% BSA at 37°C for 2 h. Next, supernatant samples or standard human IgA1 (Calbiochem, La Jolla, CA, USA) were added to each well (100 µl) and incubated at 37°C for 2 h. The plates were washed five times, then biotin-labeled lectin Vicia (Vector Laboratories Associates, USA) was added to each well (100 µl) and incubated at 37°C for 1 h. The plates were washed five times, then HRP-labeled streptavidin (1:4,000, Beyotime Institute of Biotechnology) was added to each well (100 μl) and incubated at 37°C for 30 min. Finally, 0.1 mg/ml of TMB was added at room temperature for 5 min. The OD was measured at 450 nm. Each sample was assayed in duplicate and repeated more than three times.

### Statistical Analysis

Data were statistically analyzed using SPSS 19.0 and GraphPad Prism 5.0 software and the results are expressed as mean ± standard error (mean ± SEM) or mean ± standard deviation (mean ± SD). The count data were analyzed by the χ^2^ test and the measurement data were analyzed by one-way ANOVA or two-way ANOVA; the comparison between two groups was performed by independent sample t-tests. The correlation was analyzed by Pearson’s correlation analysis and linear regression. *p*<0.05 was considered to be statistically significant.

## Results

### miR-630 Is Decreased in Tonsil Tissue From IgA Nephropathy Patients and Correlated With Clinical Parameters

**Figure 1:**
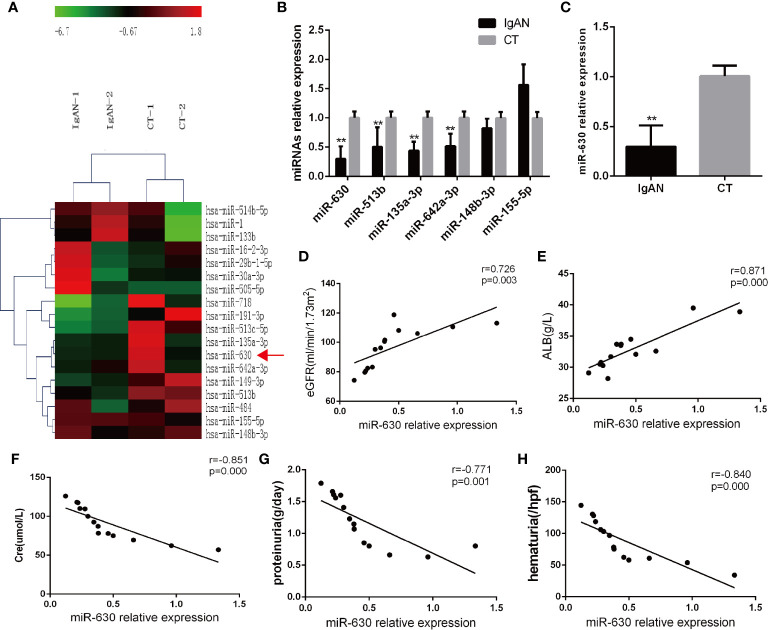
*miR-630 is decreased in the tonsil tissue of the IgAN group*. **(A)** *The microarray* analysis detected the different expression of microRNAs (miRNAs) in palatine tonsil tissue between IgAN group and CT group. N=2. **(B)** Quantitative reverse transcription-PCR (qRT-PCR) confirmed the different expression of miRNAs between IgAN group and CT group. **(C)** The expression of miR-630 was detected in tonsil mononuclear cells (TMCs) derived from the IgAN group and CT group, respectively. **(D–H)** The correlation between the expression of miR-630 and the clinical parameters including estimated glomerular filtration rate (eGFR), albumin (ALB), Cre, proteinuria, and hematuria were analyzed by Pearson correlation analysis and linear regression analysis. N=14. The data were expressed as mean ± SEM, **p < 0.01. IgAN, IgA nephropathy, N=27; CT, chronic tonsillitis, N=20.

Microarray analysis was used to detect the differential expression of miRNAs in palatine tonsil tissue between the IgAN group and the CT group. It was found that there were 29 differentially expressed miRNAs compared with the CT group: 7 miRNAs were up-regulated and 22 miRNAs were down-regulated. The data were shown in **Figure 1A** and **Supplemental Table 3**. The qRT-PCR results showed that miR-630, miR-513b, miR-135a-3p, and miR-642a-3p were significantly decreased in the IgAN group compared with the CT group; the decrease in the expression of miR-630 was the most significant (**Figure 1B**). Moreover, miR-630 expression was also significantly lower in TMCs derived from the IgAN group compared with those from the CT group (**Figure 1C**). Interestingly, the Pearson’s correlation analysis showed that the levels of miR-630 in tonsils were positively correlated with estimated glomerular filtration rate (eGFR) and albumin (ALB), but negatively correlated with creatinine (Cre), proteinuria, and hematuria (**Figures 1D–H**). These data suggest that the expression of miR-630 in palatine tonsils is correlated with clinical parameters and might be associated with *IgAN*.

### miR-630 Regulates the Concentration and Glycosylation Level of IgA1

**Figure 2:**
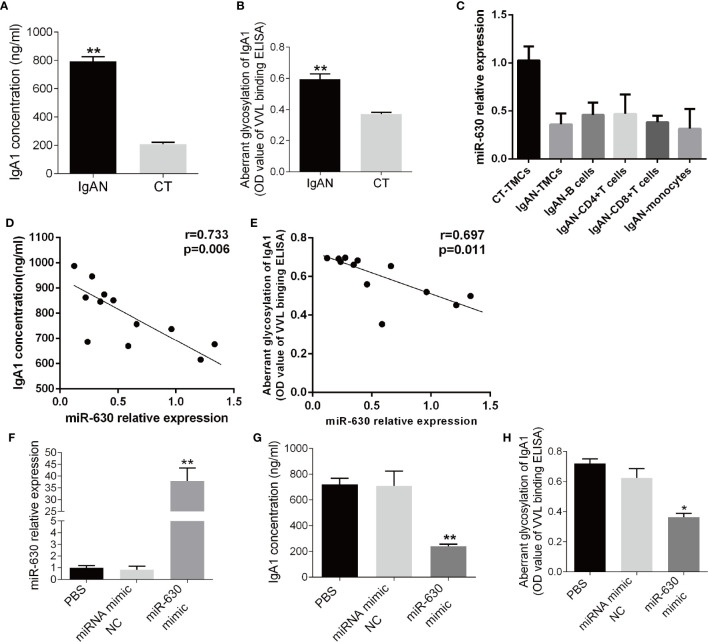
miR-630 regulates the concentration and glycosylation level of IgA1. **(A)** ELISA detected the concentration of IgA1 in the supernatant of TMCs derived from IgAN and CT group, respectively. **(B)** The binding force of IgA1 with the broad bean lectin [IgA1-*Vicia villosa* lectin (VVL)-binding OD value] was measured by enzyme-linked lectin assay. **(C)** qRT-PCR detected the expression of miR-630 in different subtypes of mononuclear cells including CD4^+^ T cells, CD8^+^ T cells, pan B cells, and pan monocytes, which were isolated from TMCs by magnetic beads. **(D, E)** The correlation between the expression of miR-630 and the concentration of IgA1 as well as the binding force of IgA1 with the broad bean lectin in TMCs were analyzed by Pearson correlation analysis and linear regression analysis. **(F)** qRT-PCR showed that the expression of miR-630 was increased significantly in TMCs transfected with miR-630 mimics. **(G, H)** overexpression of miR-630 decreased the concentration of IgA1 and the binding force of IgA1 with the broad bean lectin in TMCs significantly. The data were expressed as mean ± SEM, **p < 0.01.*p < 0.05. IgAN, IgA nephropathy, N=27; CT, chronic tonsillitis, N=20. NC, negative control.

Since the level of IgA1 is associated with the pathology of IgAN, the concentration of IgA1 in the supernatant of TMCs was measured by ELISA. It was found that the concentration of IgA1 significantly was higher in the IgAN group compared to the CT group (**Figure 2A**). Additionally, the binding force of IgA1 with broad bean lectin (IgA1-VVL-binding OD value) was much higher in the IgAN group, indicating that the levels of IgA1 glycosylation and secretion in TMCs in the IgAN group were greatly reduced (**Figure 2B**). Since the results of magnetic beads demonstrated that there was no significant difference in miR-630 expression in different subtypes of mononuclear cells including CD4^+^ T cells, CD8^+^ T cells, pan B cells, and pan monocytes (**Figure 2C**). We collected all of the mononuclear cells in the tonsil tissue for our further experimental study. The Pearson’s correlation analysis and linear regression analysis showed that the expression of miR-630 was negatively correlated with the concentration of IgA1 as well as the binding force of IgA1 with the broad bean lectin in TMCs (**Figures 2D, E**). Furthermore, with the overexpression of miR-630 after transfection with the miR-630 mimic in TMCs (**Figure 2F**), both the concentration of IgA1 and the binding force of IgA1 with broad bean lectin in TMCs were significantly decreased (**Figures 2G, H**). These data suggest that miR-630 may regulate the generation and low glycosylation level of IgA1.

### TLR4 Is the Target Gene of miR-630

**Figure 3:**
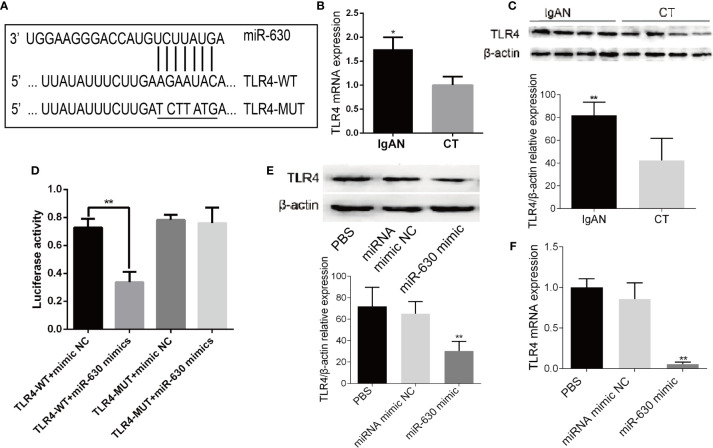
TLR4 is the target gene of miR-630. **(A)** Bioinformatic analysis showed that the miR-630 putative target sites in TLR4 3’-UTR. The mutated nucleotides were underlined. **(B, C)** qRT-PCR and western blot analysis detected the level of TLR4 mRNA and protein in the TMCs derived from the IgAN group and CT group, respectively. **(D)** The WT-TLR4 3’-UTR and the MUT-TLR4 3’-UTR reporters were co-transfected with miR-630 mimic or negative control into TMCs. Forty-eight hours after transfection, the luciferase activities were measured. **(E, F)** TMCs were transfected with negative miRNA mimics or miR-630 mimic respectively and harvested for the examination of TLR4 protein and messender RNA (mRNA) by qRT-PCR and western blot analysis. The data were expressed as mean ± SEM, *p < 0.05, **p < 0.01. IgAN, IgA nephropathy; CT, chronic tonsillitis. NC, negative control.

In order to explore the mechanism by which miR-630 regulates the concentration and glycosylation level of IgA1, we used TargetScan, miRanda, and PicTar to predict the target genes of miR-630. Some of the common predicted target genes and the gene ontology (GO) enrichment analysis, which were shown in **Supplemental Figure 1** and **Supplemental Table 4**. Among the predicted target genes, we found that TLR4 was one of the putative targets of miR-630 (**Figure 3A**). Interestingly, qRT-PCR and western blot analysis verified that the expression of TLR4 was significantly increased in TMCs derived from the IgAN group compared with the CT group (**Figures 3B, C**). Furthermore, a luciferase reporter construct containing the wild-type (WT) or mutant 3’-UTR coding sequences for TLR4 was introduced with the miR-630 mimic into TMCs, and the overexpression of miR-630 significantly decreased the relative luciferase activity of the WT-3’-UTR of TLR4 reporter plasmids, but the inhibitory effect of miR-630 on the relative luciferase activity was abrogated after the TLR4 mRNA 3’-UTR was mutated. Scramble miRNAs mimic, however, had no effect on the WT or mutant constructs (**Figure 3D**). Moreover, overexpression of miR-630 significantly reduced both the level of TLR4 protein and mRNA (**Figures 3E, F**). These results suggest that TLR4 is the predicted target gene of miR-630.

### TLR4 Is Associated With the Concentration of IgA1 and the Glycosylation Level of IgA1

**Figure 4:**
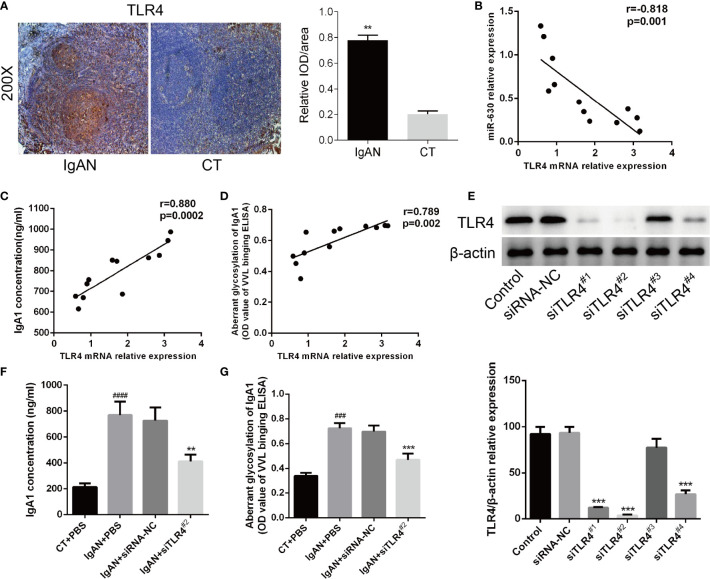
TLR4 is involved in miR-630 regulating the concentration and glycosylation levels of IgA1 in IgAN. **(A)** Semi-quantitative analysis of immunohistochemistry staining showed the expression of TLR4 in the IgAN group and CT group. Representative images were shown. N=5. **(B)** The expression of TLR4 mRNA was negatively correlated with miR-630 in the TMCs derived from the IgAN group. **(C, D)** TLR4 mRNA expression was positively correlated with the concentration of IgA1 and the binding force of IgA1 with the broad bean lectin. **(E)** The validity of TLR4 small interfering RNAs (siRNAs) was verified by western blot analysis. **(F, G)** Knocking down the expression of TLR4 made the concentration of IgA1 and the binding force of IgA1 with the broad bean lectin decreased significantly in TMCs derived from IgAN. The data were expressed as mean ± SEM, **p < 0.01.***p < 0.001, compared with siRNA-NC; ^###^p < 0.001, ^####^p < 0.0005, compared with CT. IgAN, IgA nephropathy; CT, chronic tonsillitis; NC, negative control.

Then we analyzed the correlation between TLR4 and IgA1 and immunohistochemistry for TLR4 expression in the germinal centers of tonsils. We observed a small, scattered distribution of TLR4 expression in reticular epithelial tissue and at germinal center margins both in the IgAN group and the CT group, but the expression of TLR4 in the IgAN group was significantly higher than that in the CT group (**Figure 4A**). Moreover, Pearson’s correlation and linear regression analysis showed that TLR4 mRNA was negatively correlated with the level of miR-630 in TMCs derived from the IgAN group (**Figure 4B**). TLR4 mRNA was positively correlated with the concentration of IgA1 and the binding force of IgA1 with broad bean lectin (**Figures 4C, D**).

The western blot analysis showed that siTLR4^2#^ knocked down the expression of TLR4 most effectively (**Figure 4E**). Therefore, we chose siTLR4^2#^ for further study; the results show that, by knocking down the expression of TLR4, both the concentration of IgA1 and the binding force of IgA1 with broad bean lectin were significantly reduced compared to the small interfering RNA (siRNA) negative control in IgAN (**Figures 4F, G**). These results indicate that TLR4 is involved in miR-630 regulation of the concentration and glycosylation levels of IgA1 in IgAN.

### TLR4 Might Regulate the Concentration of IgA1 and the Glycosylation Level of IgA1 Through NF-κB Signaling Pathway

**Figure 5:**
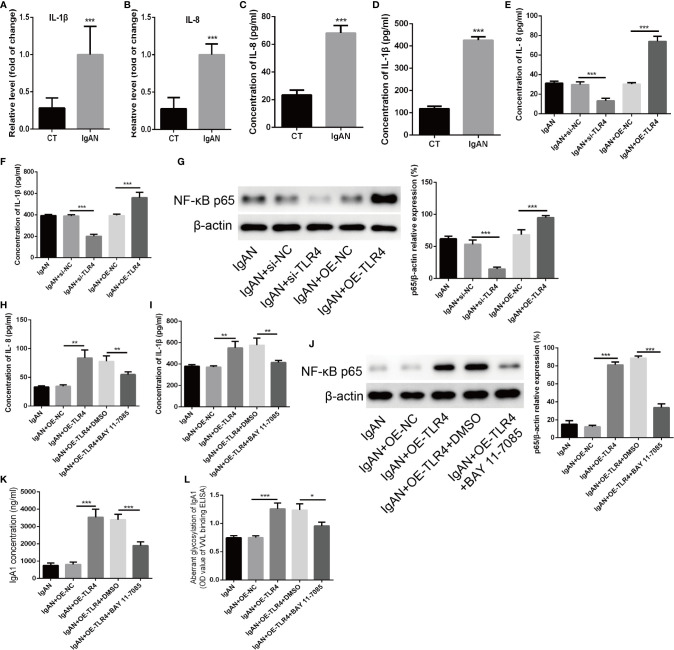
TLR4 might regulate the concentration of IgA1 and the glycosylation level of IgA1 through NF-κB signaling pathway. **(A, B)** The expressions of IL-1β and IL-8 were detected by RT-PCR in TMC from the IgAN group and CT group. **(C, D)** The concentrations of IL-1β and IL-8 were measured by ELISA in TMC from the IgAN group and CT group. **(E, F)** The concentrations of IL-11β and IL-8 were measured by ELISA when knocking down the expression of TLR4 by using TLR4 siRNA or overexpression of TLR4 in TMC. **(G)** The level of NF-κB p65 was measured by western blot analysis when knocking down the expression of TLR4 by using TLR4 siRNA or overexpression of TLR4 in TMC. **(H, I)** The effect of TLR4 on the concentration of IL-1β and IL-8 were measured by ELISA when blocking the NF-κB p65 signaling pathway by using BAY 11-7085. **(J)** The effect of BAY 11-7085 on inhibiting NF-κB p65 signaling pathway was tested by western blot analysis. **(K, L)** The effect of TLR4 on the concentration of IgA1 and the glycosylation level of IgA1 were abolished by blocking the NF-κB p65 signaling pathway. The data were expressed as mean ± SD. *p < 0.05. **p < 0.01.***p < 0.001. IgAN, IgA nephropathy; CT, chronic tonsillitis; NC, negative control.

Finally, The possible mechanism of TLR4 regulating the concentration of IgA1 and the glycosylation level of IgA1 was explored. Firstly, the expressions of IL-1β and IL-8 detected by RT-PCR as well as the concentration of that measured by ELISA were increased significantly in TMC from IgAN group compared with that from CT group (**Figures 5A–D**). When knocking down the expression of TLR4 in TMC, the results demonstrated that the levels of IL-1β and IL-8 were decreased significantly and overexpression of TLR4 got the opposite results (**Figures 5E, F**). However, different expressions of TLR4 had no significant influence on both the expression of C1GALT1 and Cosmc while both the mRNA and protein level of those were increased significantly in TMC from IgAN group (**Supplemental Figure 2**). Moreover, knocking down the expression of TLR4 could block the NF-κB p65 signaling pathway (**Figure 5G**). The last but not the least, we overexpressed TLR4 accompanied by using NF-κB p65 inhibitor BAY 11-7085 to block the NF-κB p65 signaling pathway (**Figure 5J**) and the results showed that inhibiting NF-κB p65 could abolish the effect of TLR4 on the concentration of IL-1β and IL-8 (**Figures 5H, I**). Furthermore, TLR4 regulating the concentration of IgA1 and the glycosylation level of IgA1 was also abolished by blocking the NF-κB p65 signaling pathway (**Figures 5K, L**). Taken together, we supposed that TLR4 might regulate the expression of IL-1β and IL-8 through NF-κB signaling pathway to modulate the concentration of IgA1 and the glycosylation level of IgA1.

## Discussion

In the present study, we found that miR-630 was significantly decreased in the tonsil tissue of IgAN patients and could regulate the generation and low glycosylation level of IgA1. Moreover, the levels of miR-630 were correlated with clinical parameters, including eGFR, Cre, proteinuria, hematuria, and ALB. The mechanistic study demonstrated that TLR4 was a direct target of miR-630 and was related to the concentration of IgA1 and the glycosylation level of IgA1 by affecting the level of IL-1β and IL-8 through NF-κB signaling pathway, suggesting that miR-630 regulates the concentration and glycosylation levels of IgA1 in IgAN in a TLR4-dependent manner.

IgAN is recognized as the most common primary glomerular disease in the world, and low IgA1 glycosylation is currently recognized as a key pathogenic factor for IgAN (18, 19). The main pathological characteristic is immune complexes formed by the deposition of IgA1 and underglycosylated IgA1 aggregates in the mesangial area (4). The low glycosylation level of IgA1 produced by the palatal tonsils of nephrotic patients is highly consistent with the IgA1 deposited in the kidney and suggests that IgA1 may be a mucosal secretion (20). Recently, a large number of studies have shown that underglycosylated IgA1 deposited in the mesangial area of IgAN was partially produced by the tonsils (21, 22). Maeda et al. demonstrated that palatal tonsillectomy resulted in better clinical remission in patients with IgAN in a long-term follow-up study (23). Studies from Asia, mainly from Japan (24) and China (25), had suggested that tonsillectomy had a positive effect on IgAN treatment. Tonsillectomy, as an adjuvant or independent treatment, could induce clinical remission in IgAN patients and reduce the incidence of ESRD. In IgAN patients treated by tonsillectomy, renal signs improved (26), hematuria and proteinuria decreased, renal prognosis improved (27), and renal damage progressed more slowly. The drawback was that there are some uncontrolled retrospective studies in the literature, which often use tonsillectomy in combination with steroids or immunosuppressants, making it difficult to assess the role of tonsillectomy alone in IgAN treatment. Compared with Asian studies, the European literature suggests that tonsillectomy has no effect on IgAN prognosis (28). A randomized controlled trial showed that tonsillectomy was not effective in treating IgA nephropathy (29). The controversy over tonsillectomy in the treatment of IgAN in Asia and Europe needs to be confirmed by further randomized controlled trials considering regional and ethnic associations. Another study also reported that palatal tonsillectomy not only improved urinalysis abnormalities, but also led a decrease in the intensity of IgA deposition in the mesangial area in IgAN patients (30). These studies all showed that abnormal immunity in the palatal tonsil mucosa in IgAN patients is involved in the incidence and progression of IgAN. In accordance with this research, we also found that the concentration of IgA1 increased significantly, while glycosylated IgA1 was greatly reduced in IgAN patients compared to CT patients. Therefore, s better understanding of IgAN and dysregulation of the tonsil immune response may help to elucidate the mechanism of IgA-induced hypoglycosylation of IgA1 in IgAN.

Recently, miRNAs have been shown to be important regulators in different renal diseases, such as renal cell carcinoma and IgAN (14, 31, 32). Moreover, miRNAs have been found to play an important role in the regulation of the immune system and immune cells selectively (33). Although some miRNAs, including miR-133a, miR-133b and miR-185, have been associated with the incidence of IgAN (34), scarcely any study has investigated miRNAs in the tonsils in IgAN. Furthermore, the molecular mechanism of IgA regulation remains unclear. We used microarray analysis to detect the differential expression of miRNAs in the tonsils of IgAN patients and CT patients, and found that the expression of miR-630 was significantly decreased both in the tonsil tissues and TMCs derived from the IgAN group. Although previous studies have reported that miR-630 functions as a tumor oncogene in renal cell carcinoma and could be regarded as a prognostic molecular signatures in renal cell carcinoma (32, 35), we found that miR-630 also played a crucial role in the pathology of IgAN. With miR-630 overexpression, the concentration of IgA1 and the binding force of IgA1 with broad bean lectin in TMCs were significantly decreased and negatively correlated with the concentration of IgA1 as well as the binding force of IgA1 with broad bean lectin in TMCs. Moreover, the levels of miR-630 in tonsils were positively correlated with eGFR and ALB, but negatively with Cre, proteinuria, and hematuria, which indicates that miR-630 was closely related to kidney function and might be regarded as a potential biomarker for the diagnosis and prognosis of IgAN. Nevertheless, the role of miR-630 in the tonsil in modulating the function of IgAN *in vivo* needs further study.

It is well-known that miRNAs usually regulate gene expression by binding to the 3’-UTR site of their target genes (36, 37). TLR4 is part of the TLR family and plays a central role in the regulation of the host immune system. Numerous studies have confirmed that TLR4 is expressed in cells secreting IgA and could increase the production of IgA and secretory IgA transport (38, 39). For instance, TLR4 expression is up-regulated in the peripheral blood mononuclear cells of patients with IgAN, particularly in association with proteinuria and heavy microscopic hematuria (40). Furthermore, our previous study also demonstrated that TLR4 activity was significantly increased in IgAN (41). In the present study, we confirmed that TLR4 was a direct target gene of miR-630 and was involved in miR-630 regulation of the concentration and glycosylation levels of IgA1 in IgAN. The evidence are as follows: firstly, the luciferase reporter confirmed that miR-630 directly targeted the TLR4 mRNA 3’-UTR. Secondly, overexpression of miR-630 significantly reduced the mRNA and protein levels of TLR4. Thirdly, TLR4 was significantly increased both in the tonsils and TMCs derived from IgAN patients and negatively correlated with the level of miR-630. Finally, TLR4 mRNA was positively correlated with the concentration of IgA1 and the binding force of IgA1 with broad bean lectin. Most importantly, we used TLR4 siRNA to directly knock down the expression of TLR4 and found that the concentration of IgA1 and the binding force of IgA1 with broad bean lectin were significantly reduced, which further verified that TLR4 is involved in regulating the concentration and glycosylation levels of IgA1 in IgAN. Of course, whether miR-630 regulates the concentration and glycosylation levels of IgA1 in an TLR4-dependent manner *in vivo* needs further study.

Till now, there were few reports on the concentration and glycosylation levels of IgA1 regulated by TLR4 so that we chose the markers affecting the expression/secretion/glycosylation of IgA to detect their expression in TMC from IgAN and CT group. The results showed that the levels of C1GALT1, Cosmc, BAFF, BAFF-R, IL-1β, and IL-8 were increased significantly in TMC from IgAN group compared with that from CT group. However, only the expressions of BAFF, BAFF-R, IL-1β, and IL-8 were changed rather than C1GALT1 and Cosmc when knocking down the expression of TLR4 (**Figure 5** and **Supplemental Figure 2**). Previous studies had demonstrated that the expressions of IL-1β and IL-8 could regulate the concentration of IgA and TLR4/NF-κB axis could modulate the expression of IL-1β and IL-8 (42–44). In present study, we also found that overexpression of TLR4 could active the NF-κB p65 signaling pathway. Moreover, inhibiting the expression of NF-κB p65 could abolish the effect of TLR4 on the concentration of IL-1β and IL-8 as well as the concentration of IgA1 and the glycosylation level of IgA1. Although NF-κB was reported to be associated with BAFF/BAFF-R (45, 46), the role of BAFF/BAFF-R in regulating the concentration of IgA1 was still not clear. Taken together, we supposed that TLR4 might regulate the expression of IL-1β and IL-8 through NF-κB signaling pathway to modulate the concentration of IgA1 and the glycosylation level of IgA1. But the detailed mechanism of regulating the concentration of IgA1 is so complex that more experiments need to be done to clarify that in the future.

## Conclusions

In summary, our results show for the first time, to the best of our knowledge, that the expression of miR-630 in the tonsil is signiﬁcantly lower in IgAN than in CT. The results reveal the underlying mechanism by which miR-630 modulates the content of underglycosylated IgA1 by targeting TLR4 in IgAN and NF-κB signaling pathway might be involved in TLR4 modulating the concentration of IgA1 and the glycosylation level of IgA1. Taken together, our findings provide new insights into the pathogenesis of IgAN and show that miR-630 may be a non-invasive biomarker used to diagnose and predict the prognosis of IgAN.

## Data Availability Statement

The original contributions presented in the study are publicly available. This data can be found here: NCBI GEO GSE159123.

## Ethics Statement

The studies involving human participants were reviewed and approved by the Ethics Committee of Second Xiangya Hospital, Central South University. The patients and participants provided their written informed consent to participate in this study.

## Author Contributions

Y-MP conceived and designed the experiments. CL performed the experiments, analyzed the data, and prepared all the figures. M-YY, W-ZY, X-FP, and L-YH provided technical support. CL wrote the manuscript. All authors contributed to the article and approved the submitted version.

## Funding

This work was supported by the Natural Science Foundation of Hunan Province, China (Grant No. 2019JJ50887).

## Conflict of Interest

The authors declare that the research was conducted in the absence of any commercial or financial relationships that could be construed as a potential conflict of interest.

The reviewer H. L. declared a shared affiliation with the authors to the handling editor at time of review.
