# Supplementary material for: microRNA-630 Regulates Underglycosylated IgA1 Production in the Tonsils by Targeting TLR4 in IgA Nephropathy
Source: Front Immunol. 2020 Nov 26;11:563699. doi: 10.3389/fimmu.2020.563699 (PMC7725902; doi:10.3389/fimmu.2020.563699)
Supplement: Supplementary file 1 [file DataSheet_1.pdf]

Title: microRNA-630 regulates underglycosylated IgA1 production in the tonsils by targeting TLR4 in IgA nephropathy

Supplemental materials

Supplemental Table 1. Characteristics in IgAN group and CT group

	**IgAN group** (n=27)	**CT group** (n=20)
Age (years)	27.5 ± 4.5	21.2 ± 9.6*
Gender (male/female)	12/15	13/7
GFR (mL/min/1.73 m^2)	96.5 ± 22.3	133.1 ± 35.7
Cre (umol/L)	91.5 ± 34.5	77.3 ± 20.1*
Hematuresis (/hpf)	89.4 ± 55.2	N.D.
PRO (g/day)	1.2 ± 0.6	N.D.
ALB (g/L)	32.8 ± 6.7	40.2 ± 5.5*

Abbreviation: GFR: glomerular filtration rate Cre: creatinine; PRO: urine protein; ALB: albumin; hpf: high power field; N.D.: non-detected. *$p<0.05$, compared with IgAN group.

Supplemental Table 2. The primer sequences of the genes for RT-PCR

Genes	Forward primer (5′-3′)	Reverse primer (5′-3′)
C1GALT1	GACCCTGAAGAACCCATTTACTT	TATCCTGCTCCTCCACTCAT
COSMC	GAAGATGCTGATGGAAAAGATG	CCTGGTTGGGGTGATAAGTC
BAFF	CCACAGAAAGGGAGCAGTCAC	TGGGAGGATGGAAACACACT
BAFF-R	CCCTGGACAAGGTCATCATT	TCTTGGTGGTCACCAGTTCA
IL-1β	GGTGTTCTCCATGTCCTTTGTA	GCTGTAGAGTGGGCTTATCATC
IL-8	CTGGCCGTGGCTCTCTTG	CCTTGGCAAAACTGCACCTT
GAPDH	GGAGCGAGATCCCTCCAAAAT	GGCTGTTGTCATACTTCTCATGG

Supplemental Table 3. Differential expression of miRNAs in tonsil tissue between IgAN group and CT group IgAN patients

miRBase accession number	miRNA Name	Fold change (IgAN/CT)
MIMAT0004518	hsa-miR-16-2-3p	2.100056477
MIMAT0015087	hsa-miR-514b-5p	2.622173944
MIMAT0004514	hsa-miR-29b-1-5p	2.808752111
MIMAT0000088	hsa-miR-30a-3p	3.475058413
MIMAT0000416	hsa-miR-1	3.735226739
MIMAT0000770	hsa-miR-133b	4.598821713
MIMAT0004776	hsa-miR-505-5p	13.452062
MIMAT0012735	hsa-miR-718	0.032664299
MIMAT0001618	hsa-miR-191-3p	0.046895733
MIMAT0005789	hsa-miR-513c-5p	0.056891883
MIMAT0004609	hsa-miR-149-3p	0.181051182
MIMAT0004595	hsa-miR-135a-3p	0.208670017
MIMAT0003299	hsa-miR-630	0.222205853
MIMAT0005788	hsa-miR-513b	0.317318002
MIMAT0004957	hsa-miR-760	0.363575708
MIMAT0020924	hsa-miR-642a-3p	0.402214386
MIMAT0002174	hsa-miR-484	0.488095418
MIMAT0005942	hsa-miR-1288	0.03506508
MIMAT0005573	hsa-miR-1225-3p	0.072123508
MIMAT0022496	hsa-miR-5703	0.096922813
MIMAT0018981	hsa-miR-4459	0.209741136
MIMAT0019213	hsa-miR-3162-3p	0.276249476
MIMAT0018100	hsa-miR-3676-3p	0.3398749
MIMAT0019791	hsa-miR-4697-5p	0.359808669
MIMAT0023252	hsa-miR-5787	0.385807819
MIMAT0019835	hsa-miR-4721	0.422333113
MIMAT0016906	hsa-miR-4274	0.424772775
MIMAT0019715	hsa-miR-4651	0.432839534
MIMAT0019006	hsa-miR-4478	0.459201094

Supplemental table 4. Part of predicted target genes of miR-630

Target Gene	Gene Name
TLR4	toll-like receptor 4
CD28	CD28 molecule
CD80	CD80 molecule
MAPK1	mitogen-activated protein kinase 1
PTPRC	protein tyrosine phosphatase, receptor type, C
BCL2L	BCL2-like 2
AKAP10	A kinase (PRKA) anchor protein 10
GNB1	guanine nucleotide binding protein (G protein), beta polypeptide 1
MAOB	monoamine oxidase B
HEATR3	HEAT repeat containing 3
FBXO30	F-box protein 30
PIGK	phosphatidylinositol glycan anchor biosynthesis, class K
CRISP3	cysteine-rich secretory protein 3
TFG	TRK-fused gene
PTPN22	protein tyrosine phosphatase, non-receptor type 22 (lymphoid)
TMED7	transmembrane emp24 protein transport domain containing 7
CYBB	cytochrome b-245, beta polypeptide
SP100	SP100 nuclear antigen
LYST	lysosomal trafficking regulator
POLR3A	polymerase (RNA) III (DNA directed) polypeptide A
EXO1	exonuclease 1
RAG1	recombination activating gene 1
CD226	CD226 molecule
LCP1	lymphocyte cytosolic protein 1 (L-plastin)
PTPN22	protein tyrosine phosphatase, non-receptor type 22 (lymphoid)
BCL11A	B-cell CLL/lymphoma 11A (zinc finger protein)
C5AR1	complement component 5a receptor 1

PTGER3	prostaglandin E receptor 3
DCDC2	doublecortin domain containing 2
PLAA	prostaglandin E receptor 3 (subtype EP3)
INHBB	inhibin, beta B
CYBB	cytochrome b-245, beta polypeptide
ZNF148	zinc finger protein 148
MPO	myeloperoxidase
APAF1	apoptotic peptidase activating factor 1
PLA2G4C	phospholipase A2, group IVC (cytosolic, calcium-independent)
SELE	selectin E
CXCL13	chemokine (C-X-C motif) ligand 13
PKHD1	polycystic kidney and hepatic disease 1 (autosomal recessive)
WWTR1	WW domain containing transcription regulator 1
GREM1	gremlin 1, DAN family BMP antagonist
EXO1	exonuclease 1
VTCN1	V-set domain containing T cell activation inhibitor 1
CD1C	CD1c molecule
LIF	leukemia inhibitory factor
BTLA	B and T lymphocyte associated
PAG1	phosphoprotein associated with glycosphingolipid microdomains 1
TP63	tumor protein p63
PTGER3	prostaglandin E receptor 3 (subtype EP3)

Supplemental figure 1

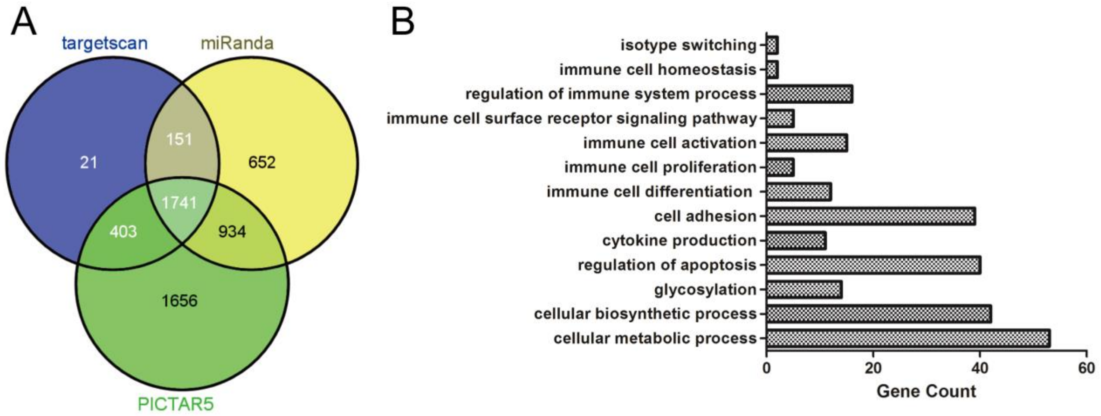

Figure S1. Bioinformatics analysis of the target genes of miR-630. (A) The common target genes of miR-630 predicted by Targetscan, miRanda and PicTar. (B) The Gene Ontology (GO) enrichment analysis of candidate target genes was conducted in DAVID database.

Supplemental figure 2

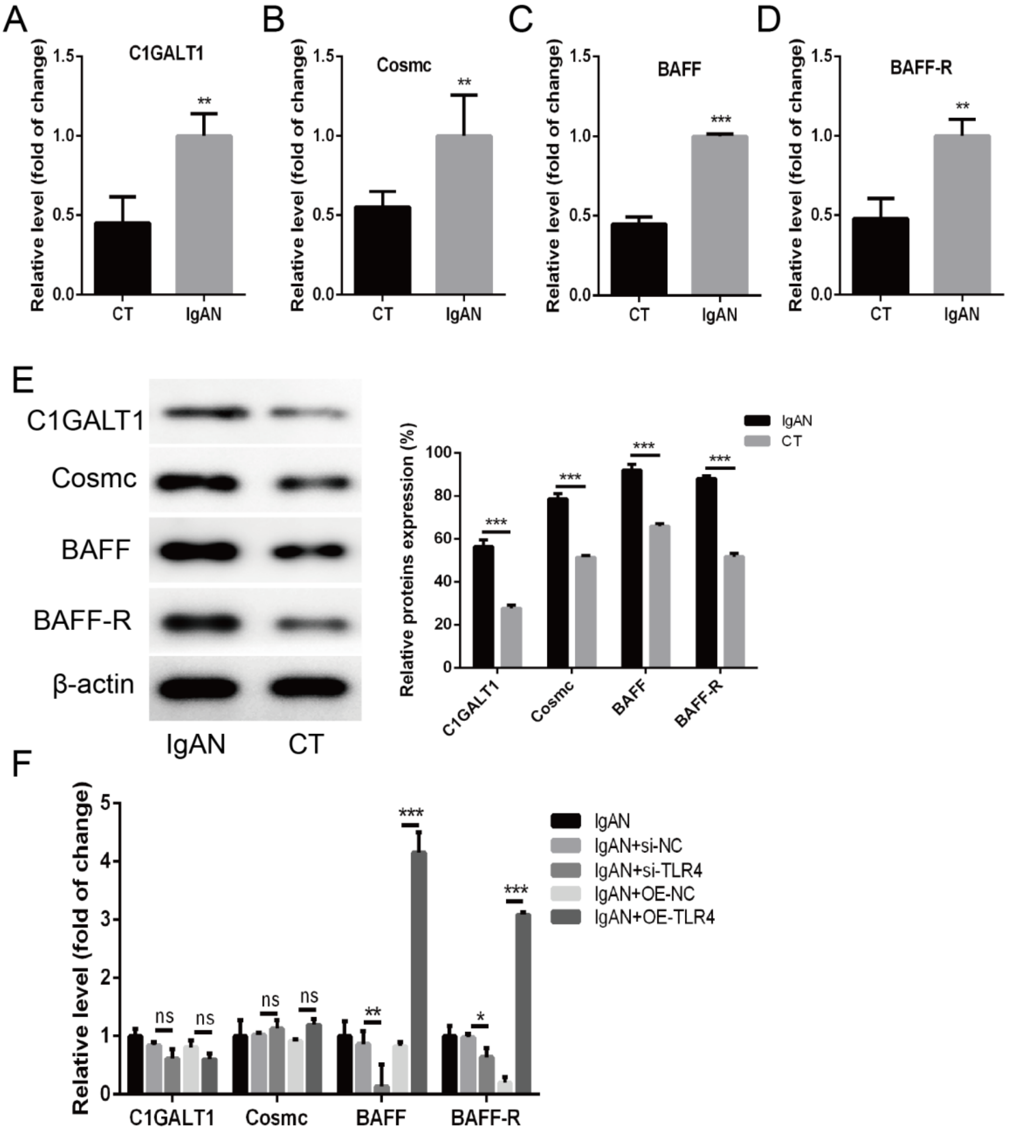

Figure S2. The expressions of related markers of affecting the expression/secretion/glycosylation of IgA. (A-D) The expressions of C1GALT1, Cosmc, BAFF and BAFF-R were detected by RT-PCR in TMC from the IgAN group and CT group. (E) The protein levels of C1GALT1, Cosmc, BAFF and BAFF-R were measured by Western blot in TMC from the IgAN group and CT group. (F) The levels of C1GALT1, Cosmc, BAFF and BAFF-R were measured by RT-PCR when knocking down the expression of TLR4 by using TLR4 siRNA or overexpression of TLR4 in TMC. The data were expressed as mean ± SD, **p < 0.01. *** p < 0.001. IgAN: IgA nephropathy; CT: chronic tonsillitis. NC: negative control. BAFF: B cell activating factor. BAFF-R: B cell activating factor receptor.
